# 24.2 NEUROCOGNITIVE PROFILES IN THE PRODROME TO PSYCHOSIS IN NAPLS-1

**DOI:** 10.1093/schbul/sby014.097

**Published:** 2018-04-01

**Authors:** Eva Velthorst, Carrie Bearden, Eric Meyer, Anthony Giuliano, Jean Addington, Kristin Cadenhead, Tyrone Cannon, Barbara Cornblatt, Thomas Mcglashan, Diana Perkins, Ming Tsuang, Elaine Walker, Scott Woods, Larry Seidman

**Affiliations:** 1 Icahn School of Medicine at Mount Sinai; 2 University of California, Los Angeles; 3 College of Medicine, College Station; 4 Worcester Recovery Center and Hospital; 5 University of Calgary; 6 University of California, San Diego; 7 Yale University; 8 The Zucker Hillside Hospital; 9 University of North Carolina; 10 Emory University; 11 BIDMC

## Abstract

**Background:**

The vast majority of studies of neuropsychological (NP) functioning in Clinical High Risk (CHR) cohorts have examined group averages, possibly concealing a range of subgroups ranging from very impaired to high functioning. Our objective was to assess NP profiles and to explore associations with conversion to psychosis, functional and diagnostic outcome.

**Methods:**

Data were acquired from 324 participants (mean age 18.4) in the first phase of the North American Prodrome Longitudinal Study (NAPLS-1), a multi-site consortium following individuals for up to 2½ years. We applied Ward’s method for hierarchical clustering data to 8 baseline neurocognitive measures, in 166 CHR individuals, 49 non-CHR youth with a family history of psychosis, and 109 healthy controls. We tested whether cluster membership was associated with conversion to psychosis, social and role functioning, and follow-up diagnosis. Analyses were repeated after data were clustered based on independently developed clinical decision rules.

**Results:**

Four neurocognitive clusters were identified: Significantly Impaired (n=33); Mildly Impaired (n=82); Normal (n=145) and High (n=64). The Significantly Impaired subgroup demonstrated the largest deviations on processing speed and memory tasks and had a conversion rate of 58%, a 40% chance of developing a schizophrenia spectrum diagnosis (compared to 24.4% in the Mildly Impaired, and 10.3% in the other two groups combined), and significantly worse functioning at baseline and 12-months. Data clustered using clinical decision rules yielded similar results, pointing to high convergent validity.

**Discussion:**

Despite extensive neuropsychological investigations within CHR cohorts, this is one of the first studies to investigate NP clustering profiles as a contributor to heterogeneity in outcome. Our results indicate that the four NP profiles vary substantially in their outcome, underscoring the relevance of cognitive functioning in the prediction of illness progression. Our findings tentatively suggest that individualized cognitive profiling should be explored in clinical settings.

